# Clinical Features of Spinal Cord Hemangioblastoma in a Dog

**DOI:** 10.3389/fvets.2015.00039

**Published:** 2015-09-30

**Authors:** Jennifer Michaels, William Thomas, Sylvia Ferguson, Silke Hecht

**Affiliations:** ^1^Department of Small Animal Clinical Sciences, College of Veterinary Medicine, University of Tennessee, Knoxville, TN, USA; ^2^Department of Biomedical and Diagnostic Sciences, College of Veterinary Medicine, University of Tennessee, Knoxville, TN, USA

**Keywords:** hemangioblastoma, intramedullary, spinal cord, tumor, dog

## Abstract

A 2-year-old male, intact Yorkshire terrier presented with a 1-month history of progressive paraparesis. Neurological examination revealed paraplegia with absent deep pain perception, decreased right pelvic limb withdrawal reflex, and lumbar pain consistent with an L4–S2 neurolocalization. Magnetic resonance imaging (MRI) showed a single, well-demarcated, intramedullary mass centered over the L3–4 disk space. A hemilaminectomy was performed, and the mass was removed en bloc. Histopathological evaluation was consistent with a hemangioblastoma. Follow-up MRI 9 months after surgery showed no evidence of tumor recurrence, and the dog was ambulatory paraparetic at that time. This case is consistent with a previous histopathological report of spinal cord hemangioblastoma in a dog and provides additional clinical information regarding diagnosis, treatment, and outcome associated with this tumor type.

## Case Presentation

A 2-year-old male, intact Yorkshire terrier [4.77 kg (10.56 lb)] was referred with a 1-month history of paraparesis starting in the right pelvic limb. Clinical signs progressed despite treatment with prednisone (2 mg/kg/day, PO). On neurological examination, the patient was paraplegic with absent deep pain perception in the pelvic limbs. The pelvic limbs had increased extensor tone, normal patellar reflexes, normal withdrawal reflex in the left, and decreased withdrawal reflex in the right. Pain was elicited on palpation of the lumbar spine. Findings were consistent with a lesion of the L4–S2 spinal cord segments.

**Figure 1:**
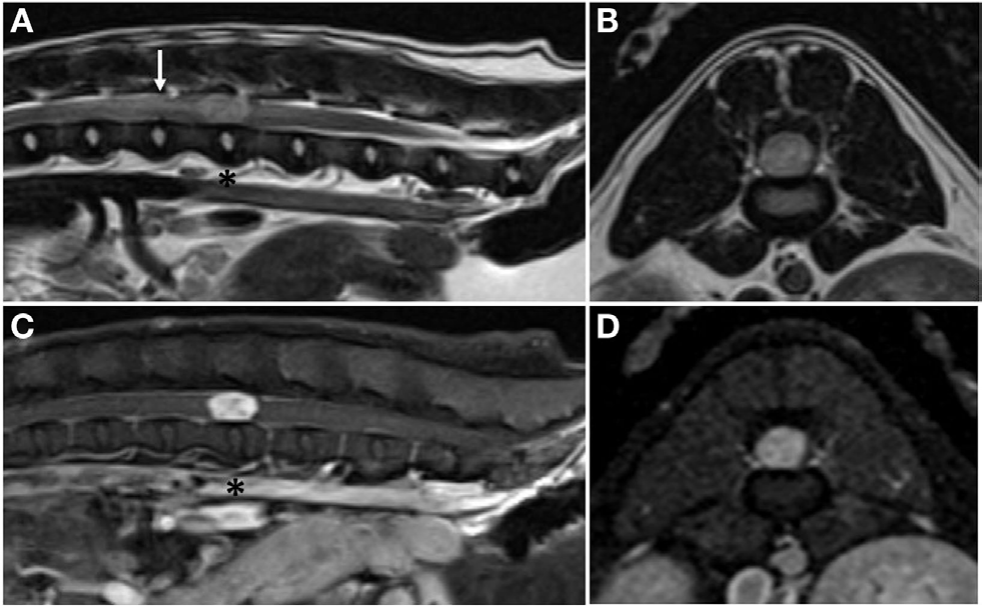
**Pre-operative magnetic resonance images**. Sagittal plane **(A)** and transverse plane **(B)** T2-weighted images showing a hyperintense, intramedullary mass at the level of the L3–4 intervertebral disk space (asterisk) and associated peritumoral edema cranial to the mass (arrow). Sagittal plane **(C)** and transverse plane **(D)** post-contrast T1-weighted images showing marked, homogenous contrast enhancement of the mass.

Complete blood count and serum biochemistry panel and electrolyte analysis were unremarkable. Magnetic resonance imaging (MRI; 1.5-T MAGNETOM Espree; Siemens) of the thoracolumbar spine revealed a slightly right-sided, discrete, intramedullary mass at the level of the L3–4 intervertebral disk space; the mass occupied over 90% of the vertebral canal (Figure 1). The mass was mildly hyperintense on T1-weighted (T1W) images, moderately hyperintense on T2-weighted (T2W), and short tau inversion recovery images, and showed intense, homogeneous contrast enhancement (Magnevist; Bayer). In addition, there was mild T2W and short tau inversion recovery hyperintensity within the spinal cord cranial to the mass, most consistent with spinal cord edema. Differential diagnoses included primary neoplasia (lymphoma, nephroblastoma, ependymoma, or glioma) and, less likely, granuloma. Thoracic radiographs were normal.

A right-sided, L3–4 hemilaminectomy was performed, and a purplish-brown, approximately 1.5-cm diameter mass was identified beneath the dura. There were prominent meningeal vessels, and following a durectomy, the mass was noticed to be highly vascularized around the margins and on the surface. Bipolar electrocautery was used to coagulate meningeal and peritumoral vessels. Initially, there was no clear plane of dissection between the mass and the surrounding spinal cord, so an ultrasonic aspirator (Sonastar XS-E, Aesculap, Center Valley, PA, USA) was used to perform the initial dissection through the pia as well as the underlying, thin, vascular tumor capsule. Once the pia and capsule were removed, a plane of dissection was easily distinguished, and the mass was removed en bloc. On further gross examination, the mass was firm and appeared noticeably more vascular along the surface than on the inside.

The dog was hospitalized and received standard post-operative care, including intravenous fluids and analgesia. At the time of dismissal from the hospital approximately 48 h after surgery, the dog was non-ambulatory paraparetic and was voiding voluntarily.

**Figure 2:**
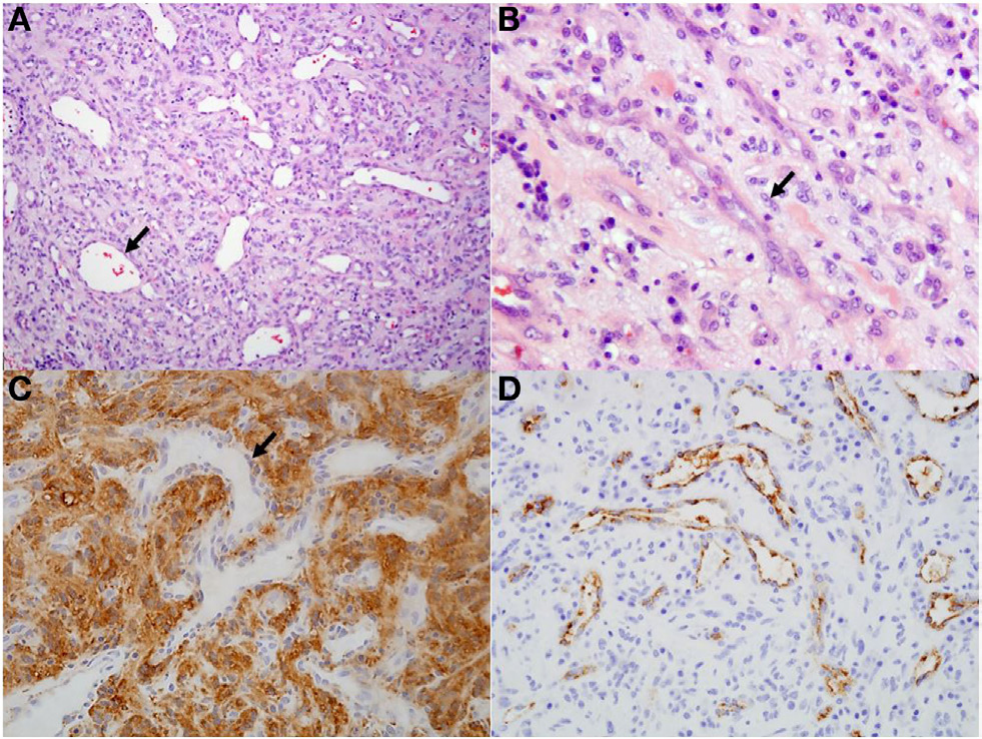
**Histological and immunohistochemical sections of spinal hemangioblastoma**. **(A)** Predominately spindle cells separated by numerous ectatic capillaries (arrow). Hematoxylin and eosin, 10×. **(B)** Higher magnification view of spindle cells (arrow) separated by moderate fibrillary stroma. Hematoxylin and eosin, 40×. **(C)** Spindle cell population and stroma within the neoplasm is diffusely positive for neuron-specific enolase. Capillaries within the section are negative (arrow; negative internal control), 20×. **(D)** Capillaries within the neoplasm are diffusely positive for factor-VIII, 20×.

On histopathological evaluation, the spinal mass was composed of mildly pleomorphic spindle cells arranged in loose streams and bundles separated by variable amounts of fibrillar stroma (Figure 2). The spindle cells had small amounts of eosinophilic cytoplasm and irregularly oval nuclei; mitoses were rare. Throughout the neoplasm were numerous, often ectatic, haphazardly arranged capillaries lined by plump endothelium. Frequently surrounding the capillaries were perivascular aggregates composed of plasma cells and fewer lymphocytes. Immunohistochemically, the spindle cells and stroma were strongly and diffusely positive for neuron-specific enolase (NSE) and negative for factor-VIII. The capillaries were diffusely factor-VIII positive and negative for NSE. These findings were consistent with a diagnosis of hemangioblastoma.

**Figure 3:**
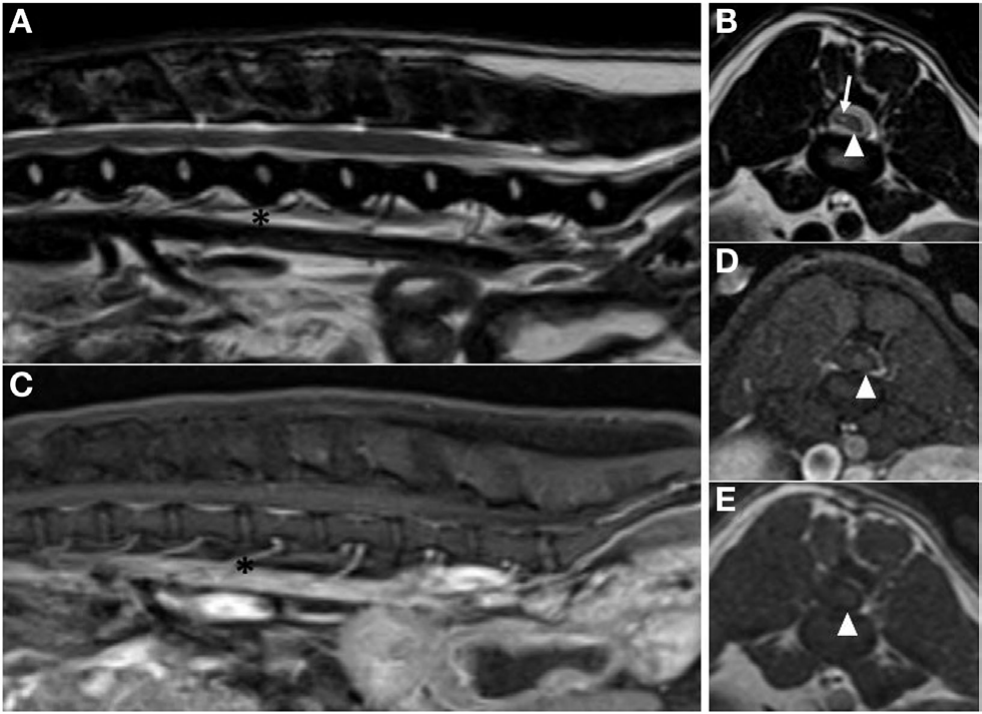
**Post-operative magnetic resonance images acquired 9 months after surgery**. **(A)** Sagittal plane T2-weighted image showing no residual peritumoral edema (asterisks are at the level of the L3–4 intervertebral disk space). **(B)** Transverse plane T2-weighted image at the level of the L3–4 intervertebral disk space, showing atrophy of the spinal cord (arrowhead). Sagittal plane **(C)** and transverse plane **(D)** post-contrast T1-weighted with Fat Sat images at the level of the L3–4 intervertebral disk space. **(E)** Transverse plane pre-contrast T1-weighted image at the level of the L3–4 intervertebral disk space. Atrophied spinal cord is visible in the left side of the vertebral canal on all transverse images (arrowhead). The right side of the vertebral canal is occupied by material that is hypointense to spinal cord on pre-contrast and post-contrast T1-weighted transverse images and mixed intensity on T2-weighted transverse image (arrow). This material may represent CSF and adhesions at the site of surgery.

The dog was re-evaluated at our institution 9 months after surgery. Neurological examination revealed ambulatory paraparesis and proprioceptive ataxia in the pelvic limbs with the right limb being more severely affected. Postural reactions in the pelvic limbs were normal on the left and delayed to absent on the right. Spinal pain was not appreciated. Follow-up MRI performed 9 months after surgery showed no evidence of tumor recurrence; however, spinal cord atrophy was present at the level of the excised hemangioblastoma. Hypointense material on pre-contrast T1-weighted and post-contrast T1W with Fat Sat images was visible to the right of the atrophied spinal cord and corresponded to an area of mixed intensity on the T2W images. This material may represent CSF and adhesions within the vertebral canal secondary to atrophy of the spinal cord and surgery (Figure 3). The application of a chemical Fat Sat to the post-contrast T1W image (but not to the pre-contrast T1W image) made distinction of this tissue from the spinal cord more apparent.

Best veterinary care was practiced in the clinical and diagnostic evaluation as well as treatment of this patient, and the owner of the patient provided informed consent for all procedures prior to them being performed. As no experimental protocols were utilized, an institutional review was neither required nor performed.

## Background

Hemangioblastomas are rare, benign, highly vascular central nervous system tumors. They composed <3% of all central nervous system tumors in people and occur most commonly in the caudal fossa, although 7.5–25% of people develop spinal cord lesions (1, 2). Most cases are sporadic, but 23–38% of hemangioblastomas occur as a result of Von-Hippel Lindau syndrome (VHL), a multi-system cancer disorder caused by a tumor suppressor gene defect (1, 3–5). Patients with VHL are significantly more likely to develop lesions within the spinal cord compared to those with sporadic hemangioblastomas (2).

To the best of our knowledge, only two cases of spinal hemangioblastoma (SHB) have been reported in dogs (6, 7). Furthermore, no prognostic information or long-term follow-up information is available because diagnosis was made at necropsy in both cases (6, 7). This article describes a case of SHB affecting the lumbar spinal cord in a young, adult dog with a focus on clinical features of the disease and long-term follow-up.

## Discussion

The clinical, MRI, surgical, and histopathological features of this tumor closely resemble descriptions of human hemangioblastomas, as well as those of previously reported canine cases. Approximately a quarter to a third of central nervous system hemangioblastomas in people occur as a result of VHL (4). In people with VHL, hemangioblastomas tend to be multiple, diagnosed in younger people, and occur more frequently in the spinal cord (3). Although there are a few isolated case reports in the veterinary literature regarding familial, multi-system cancer disorders, to the author’s knowledge, a mutation in the VHL gene has not been identified as a cause for these disorders in dogs, nor has there been any investigation into the possibility of a familial disorder as the cause of hemangioblastomas in dogs (8, 9).

In human patients, SHBs are significantly more likely to occur in the cervical or thoracic spinal cord (>90%) (1, 2, 4). The location of previously reported cases of SHB in dogs (C7, T1) was consistent with the human trend, whereas the SHB in the case reported herein had a mid-lumbar location, which is much less common in people (6, 7). Up to 75% of human SHBs are intramedullary, whereas about 23% are intradural extramedullary (5). The tumor in the current case was intramedullary but extended to the pial surface.

Many people with VHL-associated SHB do not have any clinical signs referable to the tumor. However, a majority of sporadically occurring SHBs are diagnosed after becoming symptomatic. In people, clinical signs caused by SHB include pain, hypoesthesia, dysesthesia, paresthesia, hyperreflexia, weakness, scoliosis, proprioceptive deficits, spasticity, and bowel and bladder abnormalities (4, 5).

Characteristic MRI features of hemangioblastoma in people include a well circumscribed, nodular to ovoid-shaped mass that is homogenously and intensely contrast enhancing. These tumors are T1W isointense or hypointense and T2W hyperintense to normal spinal cord parenchyma (10). The prevalence of tumor-associated spinal cord edema, peritumoral cyst formation, and/or syrinx formation is approximately 75% (5). Although these secondary changes can contribute to or be primarily responsible for clinical symptoms of the disease, no direct treatment is indicated, as these changes almost always resolve over time following surgical resection of the tumor (5, 10). The MRI findings in the case reported here are consistent with findings reported in people, except that the SHB reported here was hyperintense on T1W sequences. Although no syrinx or peritumoral cyst was present, an area of peritumoral edema was noted (Figure 1).

Use of radiation therapy, chemotherapy, and stereotactic radiosurgery has been reported for the treatment of SHB in people, although stereotactic radiosurgery is used primarily for treatment of intracranial hemangioblastoma. Despite these alternative treatment options, microsurgical resection is considered the treatment of choice for SHB in people, and a surgical plane of dissection typically facilitates total resection (5). Myelotomy might be required for tumors that do not reach the surface of the spinal cord (5). A specific method for resection of SHB and typical surgical findings has been described in human patients. Briefly, following laminectomy, a durectomy is performed on midline and the dura is reflected. Supplying and draining vessels crossing the margin of the tumor are coagulated, and the pia is incised, permitting development of a plane of dissection at the interface of the tumor capsule and spinal cord. The tumor is then resected circumferentially while applying gentle retraction (11). This description closely approximates the intraoperative findings and surgical technique used for the en bloc resection of the tumor reported here.

Histopathological and immunohistochemical findings in this case are consistent with previous veterinary reports of hemangioblastoma (6, 7, 12). There is also correlation with histopathological descriptions in humans, in which hemangioblastomas are described as highly cellular, vascularized, and composed of haphazardly oriented, small blood vessels lined by a single layer of plump endothelium with pleomorphic stromal cells filling the spaces between the vascular channels. Immunohistochemistry is critical in the diagnosis of hemangioblastoma in people; the stromal component is consistently immunoreactive for NSE, S-100 protein, inhibin A, and D2-40 while 100% of cases have endothelial cells with strong immunoreactivity to factor-VIII (1). This description is consistent with the immunoreactivity to NSE and Factor-VIII in this case.

In a review of all reported cases of sporadic SHB in people undergoing surgery, neurological improvement was described in 65% of patients whereas 24% remained unchanged (5). Recurrence rate for completely resected sporadic SHB is between 6.25 and 7.7% with partial tumor resection found to be the only significant risk factor for recurrence (2, 5).

## Concluding Remarks

In conclusion, hemangioblastoma should be considered as a rare differential diagnosis for dogs with intramedullary spinal cord masses. Evidence from human literature as well as experience with this case suggests that en bloc surgical resection is not only possible but may be the treatment of choice in dogs. This case also demonstrates the possibility of clinical improvement and long-term control of the disease with surgical resection alone.

## Conflict of Interest Statement

The authors declare that the research was conducted in the absence of any commercial or financial relationships that could be construed as a potential conflict of interest.
